# Supplementary figures and images for: The effects of anthropogenic disturbance and seasonality on the ant communities of Lang Tengah Island
Source: PeerJ. 2023 Oct 18;11:e16157. doi: 10.7717/peerj.16157 (PMC10590099; doi:10.7717/peerj.16157)

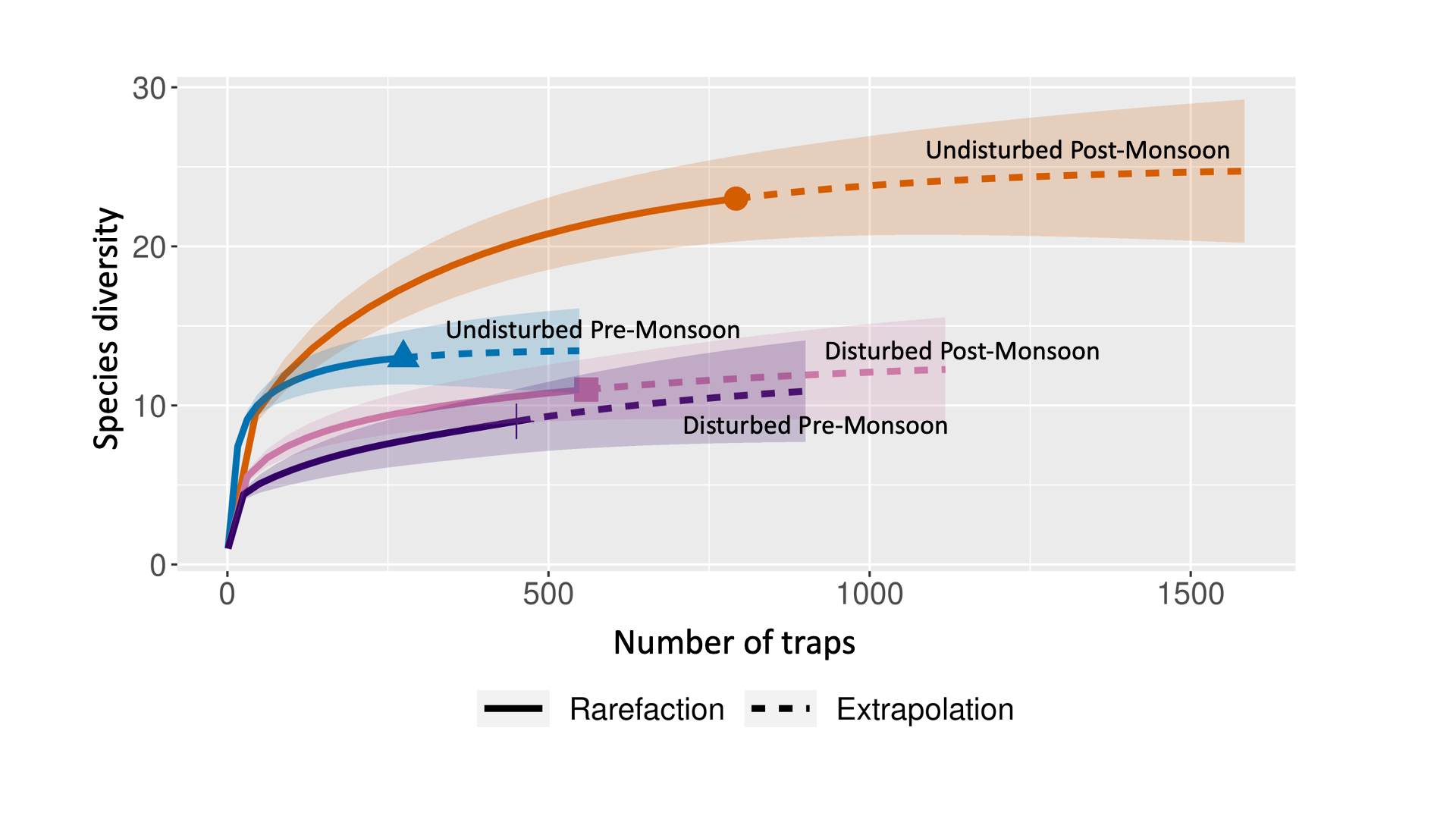

Supplement: Figure S1 — The sample-based interpolation (solid coloured lines) represents the species diversity from the actual number of pitfall traps and sample-based extrapolation (dashed coloured lines) indicates sampling completeness and the estimated species diversity if sampling effort (i.e. number of traps) were to increase. [file peerj-11-16157-s003.png]
